# Genome-Wide Identification and In Silico Characterization of the Salt Overly Sensitive 3/Calcineurin B-like (*GhSOS3*) Gene Family in Upland Cotton (*Gossypium hirsutum* L.) and Expression Profiling of Two Members Under Drought, Salt and Heat Stress

**DOI:** 10.3390/ijms27219561

**Published:** 2026-10-26

**Authors:** Laraib Iqra, Muhammad Naveed Shahid, Muhammad Nadeem Shah, Umar Akram, Dragana Božić, Imran Haider, Yuncai Hu, Muhammad Bilawal Junaid, Salah El-Hendawy

**Affiliations:** 1 Department of Botany, Division of Science and Technology, University of Education, Lahore 54000, Pakistan; laraibiqra841@gmail.com (L.I.); naveed.shahid@ue.edu.pk (M.N.S.); 2 Merrillville Education Center, Indiana Wesleyan University, 8415 Georgia St, Merrillville, IN 46410, USA; muhammadnadeem.shah@myemail.indwes.edu; 3 The Institute of Plant Breeding and Biotechnology (IPBB), MNS-University of Agriculture, Multan 60000, Pakistan; 4 Faculty of Agriculture, University of Belgrade, Nemanjina 6, 11080 Belgrade, Serbia; 5 National Research Center of Intercropping, The Islamia University of Bahawalpur, Bahawlpur 63100, Pakistan; imran.haider@iub.edu.pk; 6 Department of Biology, Nakhchivan State University, Nakhchivan 85354, Azerbaijan; 7 Precision Agriculture Laboratory, School of Life Sciences, Technical University of Munich, 85354 Freising, Germany; 8 Department of Plant Production, College of Food and Agriculture Sciences, King Saud University, P.O. Box 2460, Riyadh 11451, Saudi Arabia; bilawallljunaid@gmail.com (M.B.J.);

**Keywords:** *Gossypium hirsutum*, SOS3/calcineurin B-like protein, genome-wide identification, SOS pathway, EF-hand domain, cis-regulatory elements, qRT-PCR

## Abstract

Upland cotton (*Gossypium hirsutum* L.) is a global cash crop cultivated extensively for its high-quality fiber and oil-rich seeds. Several gene families are involved in the regulatory pathways that enable plants to acclimate to adverse abiotic stresses. One such gene family is the Calcineurin B-like (CBL)/Salt Overly Sensitive-3 (SOS3) family of calcium-sensor proteins associated with plant signaling. Although GhSOS3 has previously been associated with salt–alkali stress responses in cotton, a family level genomic characterization of SOS3-like genes in the allotetraploid *G. hirsutum* genome has not been reported. We performed in silico characterization of the *GhSOS3* gene family, including gene architecture, phylogenetic relationships, protein characterization, selection pressure, promoter elements, collinearity, chromosomal position, and protein structure modeling. This analysis identified five putative *GhSOS3* genes. The encoded proteins showed conserved EF-hand-related motifs, with differences in gene structure, predicted physicochemical properties, and motif composition. Putative abscisic acid-responsive (ABRE), salicylic acid-responsive (SARE), drought stress-responsive (DSRE) and gibberellin-responsive (GRE) elements predicted in the promoters suggest that the *GhSOS3* genes may be transcriptionally responsive to abiotic stress-related signals; however, these predictions require experimental validation. Experimentally, qPCR analysis showed stress- and time-dependent changes in the transcript abundance of *GhSOS3-2* and *GhSOS3-3* under drought, salt, and heat treatments in three cotton genotypes, indicating differential transcriptional responses to these stress conditions. Collectively, these findings establish a genomic and expression framework for future functional studies of *GhSOS3* candidates in calcium-mediated stress responses in cotton.

## 1. Introduction

*Gossypium hirsutum* is one of the most significant fiber-producing crops and an important component of the global agricultural economy [1]. As an allotetraploid crop, it possesses a complex duplicated genome, which may contribute to its adaptability to drought, heat, and salt stress [2]. However, its yield has declined in recent years under abiotic stresses [3]. Some traditional breeding methods have helped breeders mitigate drought, salt and heat stress, but only to some extent [4,5]. Abiotic stresses, including drought, heat, and salinity, have emerged as leading challenges in modern agriculture as they not only limit productivity but also compromise fiber quality in cotton [6]. Abiotic stresses directly disrupt cellular physiology, triggering ion imbalances, oxidative stress, and intracellular toxicity [7,8]. A large proportion of the world’s agricultural land is affected by drought, heat, and salinity, posing a serious threat to upland cotton fiber quality and oil content globally [3,8].

The ability of *G. hirsutum* to perceive these stressors depends on a wide web of stress-responsive genes, transporter proteins, kinases, signaling molecules, and transcription factors [9]. Hormonal signals and transcription factors regulate intricate stress-responsive pathways in cotton [10]. One of the best-characterized ion-homeostasis pathways in plants is the Salt Overly Sensitive (SOS) signal transduction pathway, components of which have also been investigated in upland cotton [11,12]. This pathway has been studied most extensively in *Arabidopsis thaliana*, where it mediates Na^+^ extrusion and contributes to the maintenance of ion balance under salinity [13]. In Arabidopsis, SOS3 (also known as Calcineurin B-like protein 4, CBL4) functions as a calcium-binding signal transducer that perceives salt-induced Ca^2+^ signals [14,15]. SOS3 interacts with the serine/threonine protein kinase SOS2 (CIPK24) to regulate the plasma membrane-localized Na^+^/H^+^ antiporter SOS1, thereby maintaining ion homeostasis [16]. The EF-hand calcium-binding motifs of SOS3 are required for calcium sensing, and the crystal structure of Arabidopsis SOS3 has provided a structural model for this mechanism [17]. SOS3/CBL4 belongs to the EF-hand calcium-sensor superfamily that acts together with CBL-interacting protein kinases (CIPKs) and ion transporters, and this module forms the core of the SOS pathway in plants [18]. Multiple homologs of *SOS3* have been identified in other crops, including *Glycine max* [19], *Brassica rapa*, *Vigna radiata*, *Vitis vinifera*, *Triticum aestivum*, *Solanum lycopersicum*, and *Hordeum vulgare*. In cotton, the CBL family has been identified at the genome level and analyzed in relation to potassium deficiency [20], and GhSOS3 has been experimentally associated with salt–alkali stress: co-expression network analysis identified a GhSOS3/GhCBL10–SOS2 module, and virus-induced gene silencing (VIGS) of *GhSOS3* and *GhCBL10* increased salt–alkali sensitivity [21]. Therefore, the involvement of GhSOS3 in stress responses in upland cotton has already been demonstrated. What has not been reported is a focused, family level characterization of the SOS3-like genes in the allotetraploid *G. hirsutum* genome that integrates their chromosomal distribution, collinearity, selection pressure, gene and protein structure, predicted promoter elements, predicted interaction partners, and stress-responsive expression in different genotypes. Unlike diploid model plants, cotton contains multiple homeologous gene copies that may have undergone subfunctionalization or neofunctionalization following polyploidization. Consequently, it remains unclear whether the *GhSOS3* gene family exhibits subgenome-specific duplication patterns, differential selective pressures among homeologous genes, or temporal divergence in stress-responsive expression [19,20]. Here, we addressed this gap by identifying five *GhSOS3* genes and characterizing them with complementary computational analyses, together with qPCR analysis of two members, *GhSOS3-2* and *GhSOS3-3*, under drought, salt, and heat stress in three cotton genotypes.

The primary aim of this research was to systematically identify and characterize the putative *GhSOS3* gene family in cotton using diverse bioinformatics methods, including the examination of conserved domains, gene architecture, conserved motifs, the predicted protein–protein interaction network, predicted subcellular localization, phylogenetic relationships, selective pressure, chromosomal distribution, collinearity, predicted phosphorylation sites and 3D structure modeling. Moreover, qPCR was used to analyze the expression of two representative genes, *GhSOS3-2* and *GhSOS3-3*, under drought, heat and salinity at specified time intervals. The novelty of this work therefore lies in the integrated, family level genomic and expression framework for *GhSOS3* genes in allotetraploid cotton, rather than in the first demonstration of a stress-related role for GhSOS3. Together, these established and predicted features provide a biological basis for investigating the *GhSOS3* gene family in cotton, while distinguishing experimentally supported observations from computationally derived predictions.

## 2. Results

### 2.1. Gene Structure, Domain, and Conserved Motif Analysis of GhSOS3

**Figure 1:**
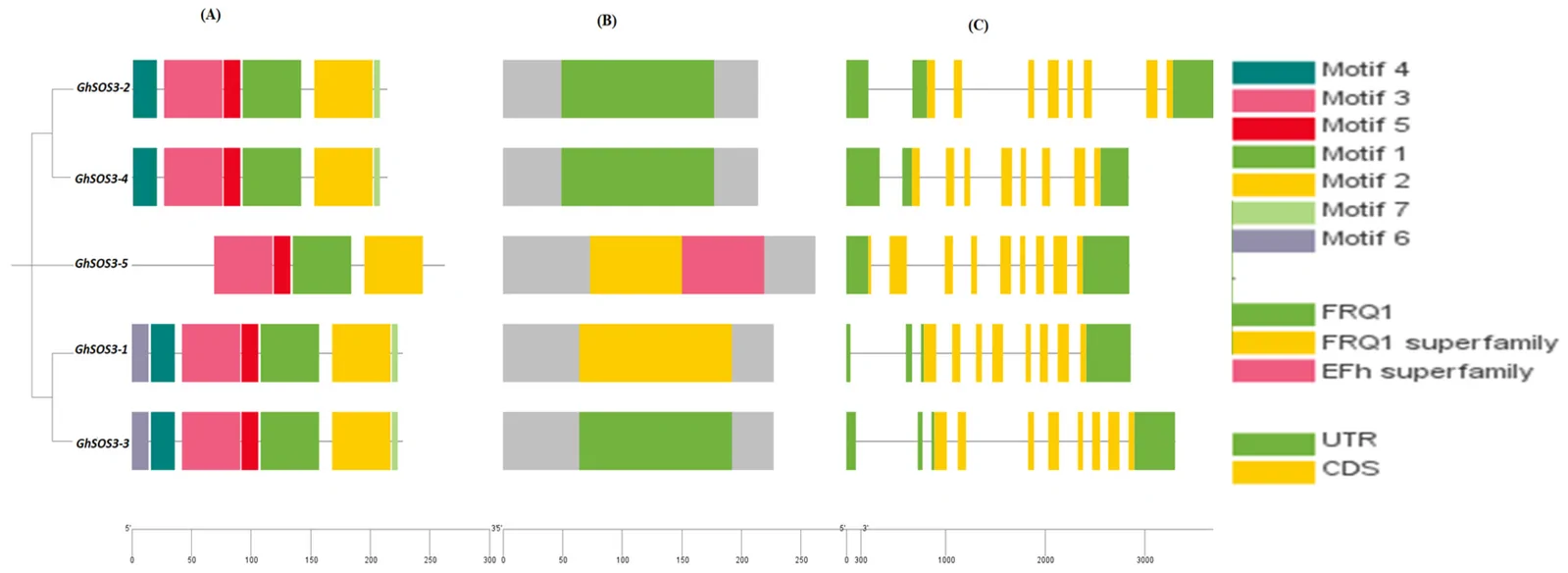
Gene structure, motif and conserved domain organization of the *GhSOS3* gene family. (**A**) Motif composition predicted by MEME (motifs 1–7); (**B**) conserved domains identified by NCBI-CDD (FRQ1, FRQ1 superfamily and EFh superfamily); (**C**) exon–intron structures (yellow boxes, CDS; green boxes, UTRs; lines, introns). The tree on the left shows the clustering of the five GhSOS3 protein sequences. Scale bars indicate amino acid positions (**A**,**B**) and gene length in bp (**C**).

Conserved domains were identified with the NCBI-CDD batch search and visualized with TBtools-II (V 2.476). NCBI-CDD detected the FRQ1 domain (Ca^2+^-binding protein, EF-hand superfamily) in GhSOS3-2 and GhSOS3-4, the FRQ1 superfamily domain in GhSOS3-1 and GhSOS3-3, and both FRQ1-superfamily and EFh-superfamily regions in GhSOS3-5 (Figure 1B). Pfam annotation identified EF-hand_7 and EF-hand_1 domains in all five proteins, with an additional EF-hand_5 domain in GhSOS3-2 and GhSOS3-4. The gene structures of the five identified genes showed variation in the number of exons and introns. The *GhSOS3* genes contained up to nine exons and eight introns, and up to seven conserved motifs were identified per protein (Figure 1A,C).

### 2.2. Protein Physicochemical Properties and Subcellular Localization of GhSOS3

**Figure 2:**
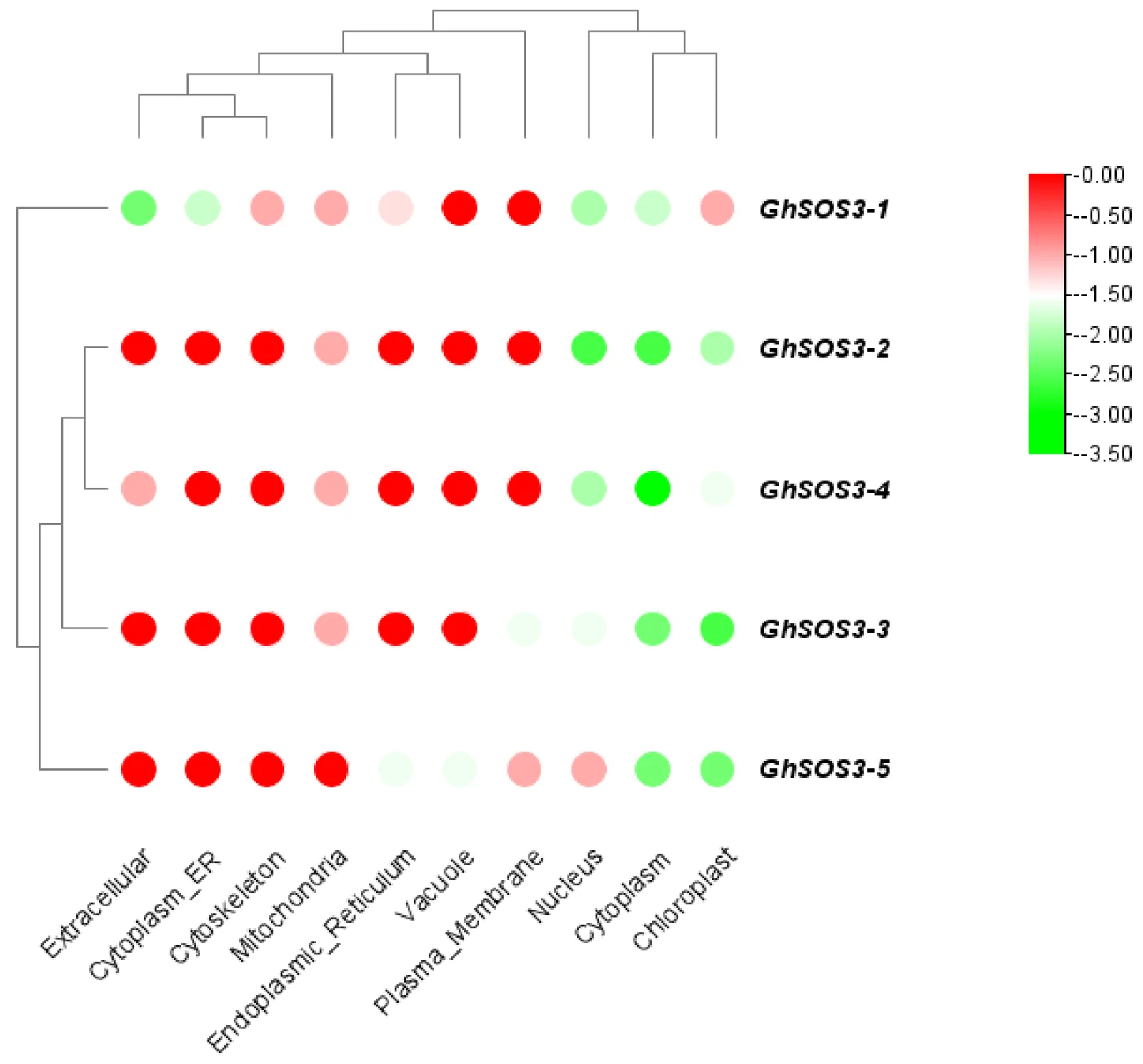
Predicted subcellular localization of *Gh*SOS3 proteins based on in silico analysis (CELLO v2.5 and WoLF PSORT). Rows represent the five GhSOS3 proteins and columns the predicted compartments; the dendrograms show hierarchical clustering of rows and columns. The colour scale shows the transformed prediction values (0 to −3.5); green denotes compartments with stronger predicted support and red denotes weaker support. These predictions require experimental validation.

*Gh*SOS3 proteins had predicted molecular weights of 24.33–30.12 kDa and lengths of 213–261 amino acids; CDS length varied from 642 to 786 bp, and predicted isoelectric points (pI) ranged from 4.72 to 4.96 (Table 1). Subcellular localization prediction indicated that the five GhSOS3 proteins differ in their predicted compartments (Figure 2). The nucleus and cytoplasm were among the compartments predicted for all five proteins; GhSOS3-1 was additionally predicted in the extracellular region, GhSOS3-2, GhSOS3-3 and GhSOS3-4 in the chloroplast, and GhSOS3-5 in the vacuole and endoplasmic reticulum. These results are computational predictions and are regarded only as hypotheses regarding the localization of *Gh*SOS3 proteins.

### 2.3. Chromosomal Mapping of GhSOS3 Genes

**Figure 3:**
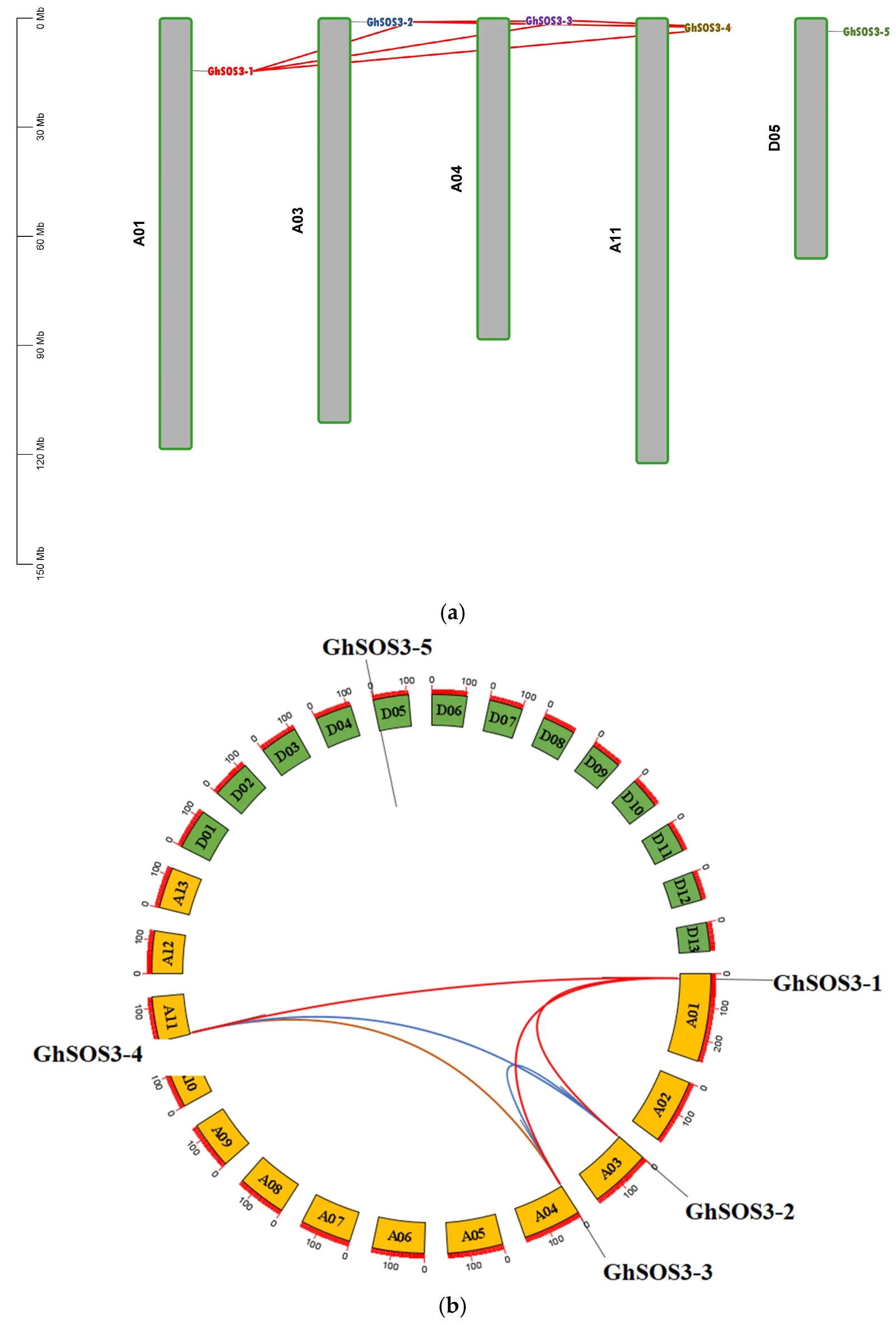
Chromosomal distribution and collinearity of *GhSOS3* genes. (**a**) Chromosomal distribution of the five *GhSOS3* genes across chromosomes A01, A03, A04, A11, and D05. Gene positions are shown according to their physical chromosomal coordinates (scale in Mb), while connecting red lines indicate collinear/syntenic relationships detected by MCScanX among *GhSOS3* loci. (**b**) Circos representation of the chromosomal localization and syntenic relationships of *GhSOS3* genes across the 26 chromosomes of the A and D subgenomes. Colored connecting lines represent syntenic gene pairs identified between chromosomes. These relationships indicate genomic conservation but do not, by themselves, establish the specific mechanism of gene duplication.

*GhSOS3* genes were located on both subgenomes (A and D): *GhSOS3-1*, *GhSOS3-2*, *GhSOS3-3*, and *GhSOS3-4* were located on chromosomes A01, A03, A04 and A11 of the A subgenome, whereas *GhSOS3-5* was located on chromosome D05 of the D subgenome (Figure 3a and Figure S2). No *GhSOS3* gene was identified on the D-subgenome homeologous chromosomes D01, D03, D04 or D11 with the search criteria applied, which may reflect gene loss, differences in genome annotation, or the stringency of the search. Thus, the identified *GhSOS3* genes showed an uneven distribution between the two subgenomes; however, this distribution alone does not provide evidence for subgenome-specific duplication bias or preferential evolutionary expansion.

### 2.4. Ka/Ks Ratios and Collinearity

Ka/Ks ratios were calculated for the six gene pairs formed among *GhSOS3-1*, *GhSOS3-2*, *GhSOS3-3* and *GhSOS3-4* (Table S2). All ratios were below one (0.018–0.134), which is consistent with purifying selection. Two pairs, *GhSOS3-1*/*GhSOS3-3* (Ks = 0.446) and *GhSOS3-2*/*GhSOS3-4* (Ks = 0.533), had relatively low Ks values, whereas the remaining four pairs had Ks values above 2.0; because synonymous sites may be saturated at such high Ks values, the corresponding Ka/Ks ratios should be interpreted with caution. Collinearity analysis with MCScanX in TBtools-II detected collinear (syntenic) relationships among *GhSOS3-1*, *GhSOS3-2*, *GhSOS3-3* and *GhSOS3-4* (Figure 3a,b), whereas no collinear partner was detected for *GhSOS3-5*. These results provide evidence of syntenic relationships among these loci; however, Ka/Ks ratios and collinearity patterns alone are not sufficient to determine the mechanism (e.g., segmental or whole-genome duplication) that generated these gene copies.

### 2.5. Phylogenetic Tree Analysis

**Figure 4:**
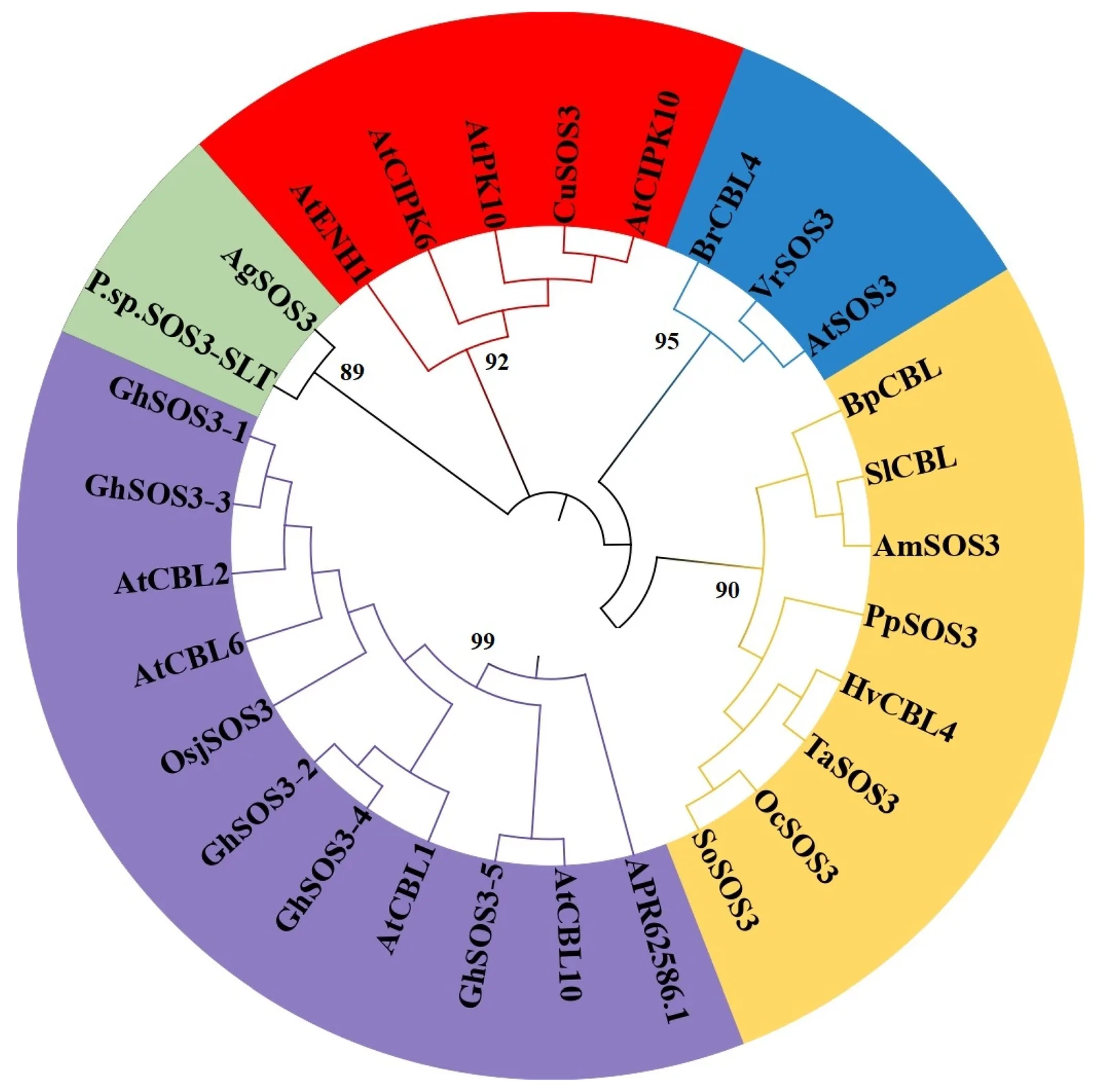
Maximum Likelihood phylogenetic tree of GhSOS3 proteins and related sequences from other plant species. The 29 amino acid sequences were aligned with ClustalW, and the tree was constructed in MEGA12 using the Jones–Taylor–Thornton (JTT) model; the final dataset comprised 780 positions. The tree with the highest log-likelihood (−13,224.85) is shown. Numbers at nodes are bootstrap support values (%) from 1000 replicates. Coloured sectors indicate the major clades. GhSOS3 proteins cluster with Arabidopsis CBL1, CBL2, CBL6 and CBL10, rice OsjSOS3 and APR62586.1, reflecting sequence-level relatedness; clustering does not by itself indicate functional equivalence. At, *Arabidopsis thaliana*; Br, *Brassica rapa*; Gh, *Gossypium hirsutum*; Hv, *Hordeum vulgare*; Oc, *Oryza coarctata*; Osj, *Oryza sativa* ssp. *japonica*; Sl, *Solanum lycopersicum*; Ta, *Triticum aestivum*; Vr, *Vigna radiata*.

To infer evolutionary relationships, a phylogenetic tree was constructed from the five GhSOS3 proteins and 24 CBL/SOS3-related and other reference sequences from different plant species (29 amino acid sequences in total; Figure 4). Sequences were aligned with ClustalW in MEGA12, and the tree was inferred by the Maximum Likelihood method using the Jones–Taylor–Thornton (JTT) substitution model, with branch support assessed from 1000 bootstrap replicates. The final dataset comprised 780 aligned positions. The five GhSOS3 proteins grouped within one clade (purple) together with Arabidopsis AtCBL1, AtCBL2, AtCBL6 and AtCBL10, rice OsjSOS3 and APR62586.1. Within this clade, GhSOS3-1 and GhSOS3-3, and GhSOS3-2 and GhSOS3-4, formed two pairs of closely related sequences, whereas GhSOS3-5 grouped with AtCBL10. Arabidopsis SOS3 (AtSOS3/CBL4) grouped in a separate clade with BrCBL4 and VrSOS3; SOS3 sequences from *Triticum aestivum* (TaSOS3), *Oryza coarctata* (OcSOS3), *Hordeum vulgare* (HvCBL4) and other species formed an additional clade, and CIPK and ENH1 sequences grouped separately. Thus, the proteins designated here as GhSOS3 are members of the broader SOS3/CBL family that are most closely related to Arabidopsis CBL1, CBL2, CBL6 and CBL10, rather than direct orthologs of AtSOS3/CBL4. The clustering of the proteins indicates evolutionary relationships at the sequence level; nonetheless, phylogenetic clustering does not imply functional equivalence among the proteins.

### 2.6. Promoter Sequence Analysis

**Figure 5:**
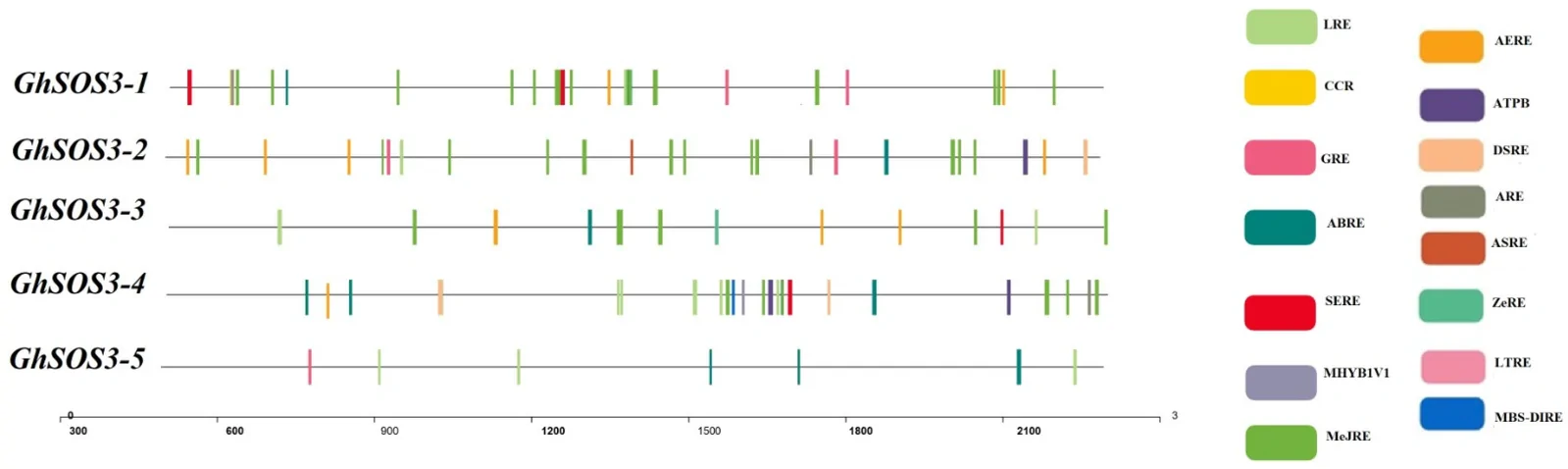
Predicted cis-regulatory elements in the 2000 bp upstream promoter regions of *GhSOS3* genes identified with PlantCARE and visualized with TBtools-II. Coloured bars indicate the positions of predicted elements: ABRE, abscisic acid-responsive element; ARE, anaerobic-responsive element; DSRE, drought stress-responsive element; GRE, gibberellin-responsive element; LRE, light-responsive element; LTRE, low-temperature-responsive element; MBS-DI, MYB binding site involved in drought inducibility; MeJRE, methyl jasmonate-responsive element; SARE, salicylic acid-responsive element; AERE, ASRE, ATBP, CCR, MYBHv1 and ZeRE, other elements as annotated by PlantCARE. The x-axis indicates position (bp) within the promoter region. The identified elements represent predicted transcription factor binding sites and should not be interpreted as experimentally validated functional regulatory regions.

Various cis-regulatory elements were predicted in the 2000 bp upstream promoter regions of all *GhSOS3* genes. These included the drought stress-responsive element (DSRE) and the MYB binding site involved in drought inducibility (MBS-DI), as well as hormone-responsive elements associated with gibberellin (GRE), abscisic acid (ABRE), methyl jasmonate (MeJRE), and salicylic acid (SARE) responses. Light-responsive (LRE), low-temperature-responsive (LTRE) and anaerobic-induction (ARE) elements, together with other predicted elements (AERE, ASRE, ATBP, CCR, MYBHv1 and ZeRE), were also identified (Figure 5). The distribution of these predicted elements indicated that the *GhSOS3* promoters contain sequence motifs potentially associated with hormonal and stress-related regulatory responses. However, these computationally identified elements do not demonstrate transcription-factor binding or regulatory activity and therefore should be considered putative regulatory features requiring experimental validation.

### 2.7. Predicted Protein–Protein Interactions, Phosphorylation Sites and 3D Structures of GhSOS3 Proteins

**Figure 6:**
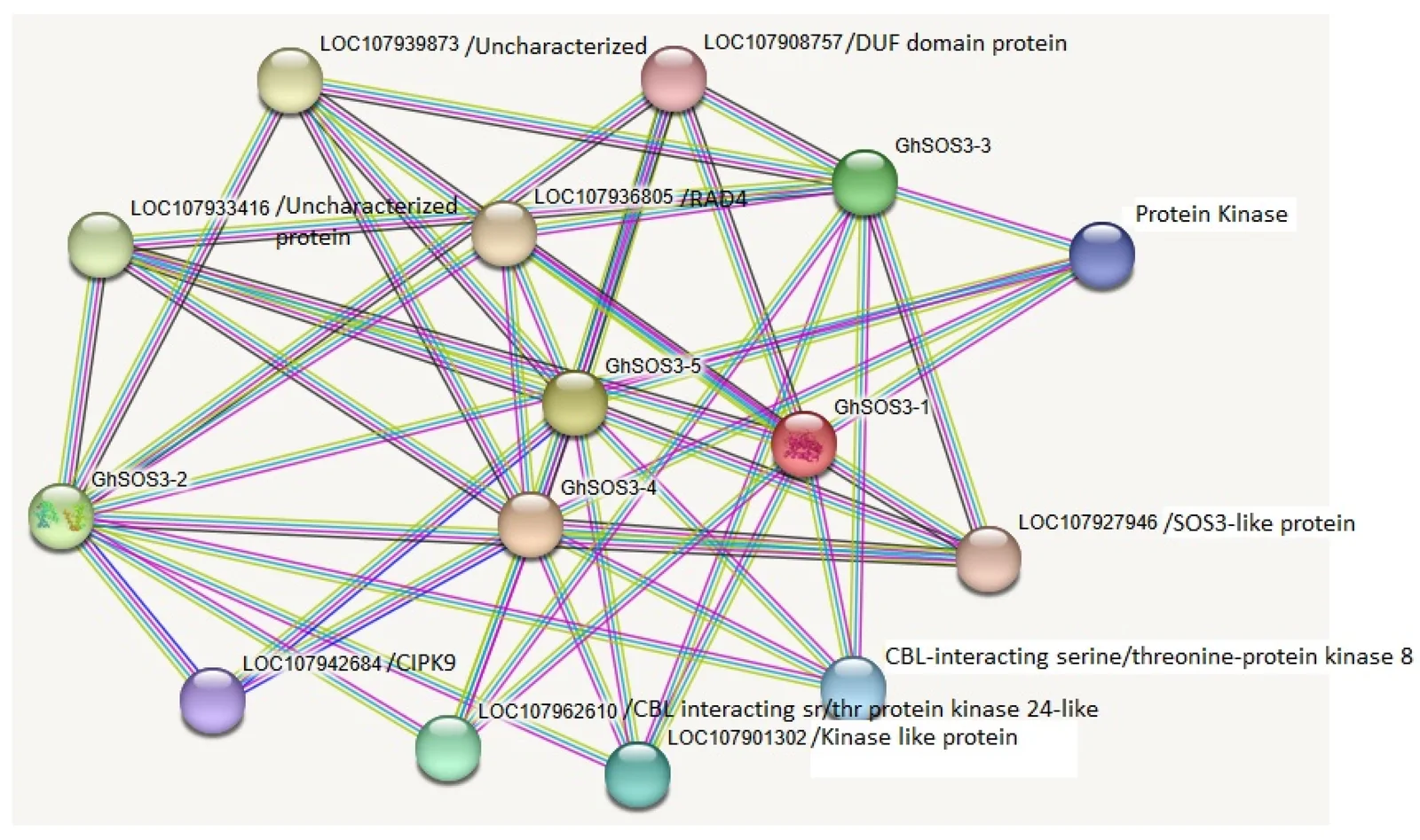
Predicted protein–protein interaction (PPI) network of GhSOS3 proteins generated with STRING (15 nodes, 48 edges). Each node is labeled with its annotated protein name; the corresponding LOC identifiers and functional annotations are listed in Table S3. GhSOS3-1 to GhSOS3-5, query proteins; CIPK8, CBL-interacting serine/threonine-protein kinase 8 (LOC107957846); CIPK9-like, CBL-interacting serine/threonine-protein kinase 9-like (LOC107942684); CIPK24, CBL-interacting serine/threonine-protein kinase 24 (LOC107901302); CIPK24-like (LOC107962610); PK, non-specific serine/threonine protein kinase (LOC107941797); RAD4, DNA repair protein RAD4 (LOC107936805); UP1–UP4, uncharacterized proteins (LOC107908757, LOC107927946, LOC107933416 and LOC107939873, respectively). Edge colours indicate the types of evidence supporting each predicted association in STRING. The network represents predicted associations and does not demonstrate physical interaction or pathway participation.

The protein–protein interaction (PPI) network predicted by STRING provides a computational framework for generating hypotheses about possible associations of GhSOS3 proteins with stress- and signaling-related proteins (Figure 6). The network comprised 15 nodes and 48 edges (PPI enrichment *p*-value < 0.1, as reported by STRING). In addition to the five GhSOS3 query nodes (annotated by STRING as CBL1-, CBL2-, CBL3- and CBL10-like proteins; Table S3), the predicted partners included CBL-interacting serine/threonine-protein kinases 8, 9-like, 24 and 24-like, a non-specific serine/threonine protein kinase, the DNA repair protein RAD4, and four uncharacterized proteins (LOC107908757, LOC107927946, LOC107933416 and LOC107939873). All nodes are labeled with their annotated protein names in Figure 6, and the corresponding LOC identifiers and functional annotations are listed in Table S3. Because several partners are uncharacterized, and because STRING associations combine evidence types such as co-expression, text mining and homology transfer, these predicted connections are regarded as candidate associations for future study, not as evidence of direct physical interaction or of the participation of GhSOS3 proteins in a particular signaling pathway, including the canonical SOS pathway.

Three-dimensional models of the five GhSOS3 proteins were predicted with AlphaFold 3, superimposed onto a common reference model so that they are displayed in the same orientation, and annotated for EF-hand motifs (EF1–EF4) and the predicted Ca^2+^-coordinating loop residues (Figure S1A). Ramachandran plots showed that most residues of the GhSOS3 models were located in the most favoured and allowed regions (Figure S1B), with residues in the right-handed α-helical and β-sheet regions predominating and only a few residues in the left-handed α-helical region. These results provide an assessment of the stereochemical quality of the predicted structural models but do not demonstrate structural stability under abiotic stress conditions. The predicted EF-hand regions indicate conserved sequence and structural features potentially associated with calcium binding; however, calcium-binding activity was not experimentally validated in this study. Phosphorylation-site prediction with NetPhos 3.1 identified the largest number of putative sites in GhSOS3-2 (14 sites above the threshold, mainly in the non-conserved terminal region), followed by GhSOS3-5 (Figure S1C). These sites represent computationally predicted post-translational modification sites and require experimental validation to establish their biological significance.

### 2.8. Expression Analysis of GhSOS3 Genes

A heatmap of publicly available RNA-seq data (BioProject PRJNA490626) showed that *GhSOS3-2* and *GhSOS3-3* had the highest overall transcript levels among the five genes across the control and stress time points, whereas *GhSOS3-5* showed the lowest levels and *GhSOS3-1* and *GhSOS3-4* showed intermediate levels (Figure S3). The highest value for *GhSOS3-3* was observed after 1 h of salt stress, whereas *GhSOS3-2* showed its highest values after 3 h of heat and 6 h of drought stress. These expression patterns provide a basis for selecting candidate genes for experimental validation but do not, by themselves, establish gene-specific biological functions or stress tolerance mechanisms.

### 2.9. qRT-PCR Expression Analysis of GhSOS3-2 and GhSOS3-3

**Figure 7:**
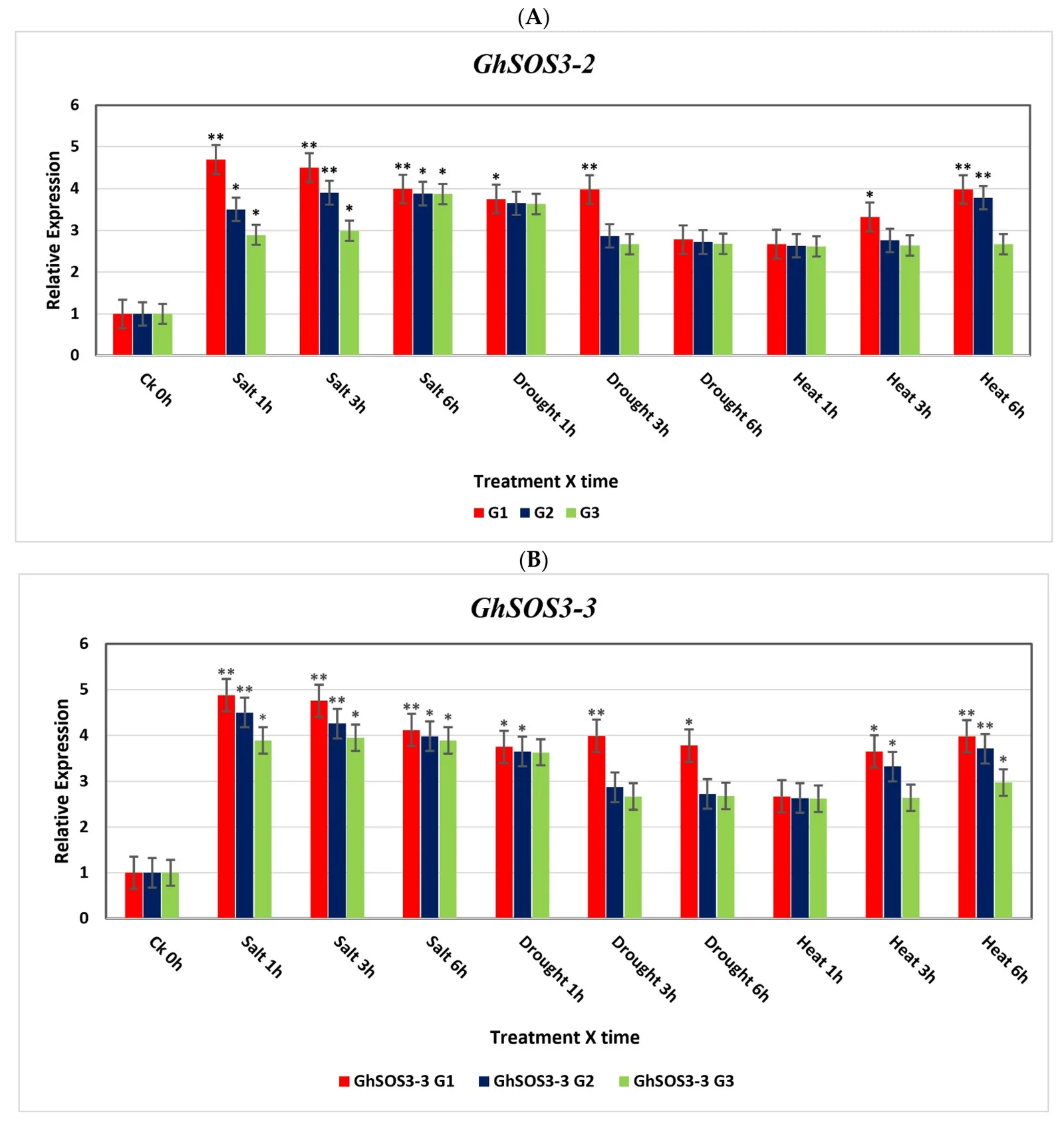
Relative expression of (**A**) *GhSOS3-2* and (**B**) *GhSOS3-3* in three cotton genotypes (G1, CIM-608; G2, MNH-886; G3, VH-295) under salt (250 mM NaCl), drought (20% PEG) and heat (45 °C) stress at 1, 3 and 6 h, determined by qRT-PCR using the 2^−ΔΔCt^ method with *GhUBQ14* as the reference gene and the 0 h control (Ck 0 h) as the calibrator. Bars represent means ± SE of three biological replicates, each analyzed with three technical replicates. Data were analyzed by three-way ANOVA (genotype × stress × time) followed by Tukey’s HSD test; asterisks (* *p* < 0.05; ** *p* < 0.01) above bars indicate statistically significant treatment- and time-specific differences.

*GhSOS3-2* and *GhSOS3-3* were selected for qRT-PCR analysis based on their higher expression levels across multiple stress conditions in the publicly available RNA-seq heatmap (Figure S3). In addition, multiple predicted stress-responsive cis-regulatory elements were present in their promoter regions; these computational features provided a basis for selecting the two genes for further expression analysis under abiotic stress conditions. Relative to the 0 h control, transcript levels of both genes were higher under all three stresses in all three genotypes (Figure 7). Of the three genotypes evaluated, genotype 1 (G1, CIM-608) consistently showed the greatest transcript abundance of both *GhSOS3-2* and *GhSOS3-3* across stress treatments and time points, genotype 2 (G2, MNH-886) showed intermediate levels, and genotype 3 (G3, VH-295) the lowest levels. These differences may reflect genotype-dependent variation in *GhSOS3* transcript abundance under the tested conditions, but the mechanisms responsible were not investigated in this study. In G1, *GhSOS3-2* transcript levels peaked after 1 h of salt stress (4.7-fold) and remained high at 3 h (4.5-fold); under drought, the highest level was observed at 3 h (4.0-fold) and under heat at 6 h (4.0-fold), whereas lower levels were recorded at 6 h of drought (2.8-fold) and 1 h of heat (2.7-fold). *GhSOS3-3* showed a similar but generally stronger response, peaking after 1 h of salt stress (4.9-fold) and remaining above 3.7-fold at all drought time points (Figure 7). Differences in the magnitude and timing of transcript accumulation between *GhSOS3-2* and *GhSOS3-3* indicate distinct expression patterns under the tested conditions; however, these patterns alone do not establish distinct biological functions or stress-tolerance roles for the two genes.

## 3. Discussion

Abiotic stresses including drought, heat, and salinity are major limiting factors for the growth, productivity, and quality of cotton fibers. These stresses affect cellular water relations, ion homeostasis, photosynthesis, and redox balance, which in turn influence plant growth [17,22,23]. Ca^2+^ signaling is one of the earliest responses of plants to different environmental stimuli. Calcineurin B-like (CBL) proteins are Ca^2+^ sensors that work as part of the CBL–CIPK signaling network [23]. This research deals with the genome-wide analysis of the *GhSOS3* gene family in *Gossypium hirsutum* from the aspects of sequence and structure, phylogeny, chromosomal location and collinearity, cis-element prediction in the promoter region, and stress-responsive expression of the putative genes. Because GhSOS3 had already been implicated in salt–alkali responses in cotton [21] and the cotton CBL family had been identified previously [20], the principal contribution of this study is the establishment of a *SOS3* family level genomic and expression framework that can guide further validation and functional investigation of *GhSOS3* candidates in cotton. A total of five *GhSOS3* genes were identified in the genome of *G. hirsutum*. All five proteins had conserved EF-hand-related motifs, supporting their classification in the larger group of CBL/SOS3-related calcium sensors. Phylogenetic analysis further showed that these proteins are most closely related to Arabidopsis CBL1, CBL2, CBL6 and CBL10 rather than to AtSOS3/CBL4 itself (Figure 4), and STRING annotated them as CBL1-, CBL2-, CBL3- and CBL10-like proteins (Table S3); the designation GhSOS3 used here therefore refers to membership of the SOS3/CBL family and does not imply that each protein is a functional ortholog of AtSOS3. EF-hand motifs are structurally related to Ca^2+^ binding in most calcium-binding proteins, and the *Arabidopsis thaliana* SOS3 protein, which is known for calcium sensing, possesses EF-hand motifs [17]. Nevertheless, the presence of EF-hand motifs in *Gh*SOS3 proteins in the current study is a sequence- and structure-based prediction, since Ca^2+^-binding activity was not tested for the cotton proteins. The conserved EF-hand motifs are consistent with, but do not demonstrate, a calcium-sensing capacity of *Gh*SOS3 proteins. The differences in motif composition among *Gh*SOS3 proteins reflect sequence diversification, but whether they affect calcium-binding properties or specificity must be determined experimentally. The *Gh*SOS3 proteins had similar predicted physicochemical features, with molecular weights and acidic isoelectric points comparable to those of CBL proteins from other plants [19,24]. The acidic nature of these proteins is compatible with the presence of negatively charged residues, which may play a role in metal binding, as reported previously [25]. However, physicochemical similarity is not enough to demonstrate biochemical activity; it indicates only that the predicted molecular features of the *Gh*SOS3 proteins are similar to those of other CBL family proteins. According to the predicted subcellular localization, the nucleus and cytoplasm were among the compartments predicted for all five *Gh*SOS3 proteins, whereas *Gh*SOS3-5 was additionally predicted in the vacuole and endoplasmic reticulum. This information should be interpreted with caution since computational predictions of subcellular localization cannot serve as experimental proof of protein localization. However, the predicted differences in subcellular localization of *Gh*SOS3 proteins may provide hypotheses for future experimental studies of the cellular distribution of this family. In Arabidopsis, SOS3/CBL proteins exhibit different subcellular localizations and are involved in different calcium-signaling pathways depending on their interacting partners and their ability to associate with membranes [25,26]. Phylogenetic analysis showed that *Gh*SOS3 proteins group together with CBL proteins of other plants, indicating their evolutionary relatedness to previously characterized members of this family. The presence of CBL-like proteins in monocots and dicots reflects their evolutionary conservation in mediating plant responses to external signals through calcium signaling [27]. Phylogenetic closeness, however, cannot confirm that individual *Gh*SOS3 proteins have the same biological function as their homologs [20,25]. Thus, functional equivalence should be regarded as a hypothesis to be tested biochemically or genetically. Evolutionary analysis revealed that the investigated *GhSOS3* gene pairs had Ka/Ks ratios below one, which is consistent with purifying selection [28]. This implies that amino acid-altering substitutions have been constrained relative to synonymous substitutions. Because four of the six pairs had Ks values above 2.0, at which synonymous sites may approach saturation, these estimates should be interpreted cautiously. Ka/Ks ratios cannot be used to establish the origin of duplication or the biological function of the gene. Collinearity analysis detected syntenic relationships among *GhSOS3-1* to *GhSOS3-4*, indicating that these loci reside in conserved genomic regions. Syntenic relationships are consistent with, but do not by themselves demonstrate, a particular duplication mechanism; distinguishing segmental, whole-genome or other duplication events would require block-level synteny analysis across *Gossypium* genomes together with dating of the duplication events. The uneven distribution of the *GhSOS3* genes between the A and D subgenomes might reflect differential retention or loss after polyploidization, or differences in genome annotation, but this possibility was not tested in the present study.

Analysis of the promoters revealed putative cis-regulatory elements associated with responses to hormones and environmental stresses, such as abscisic acid, methyl jasmonate, salicylic acid, drought, and gibberellin. These cis-acting elements have been well documented in stress-responsive plant promoters and may be target sequences for transcription factors [29]. The presence of predicted drought-associated cis-elements provides a possible regulatory context for the stress-responsive expression observed for selected *GhSOS3* genes. However, the functionality of these elements remains to be experimentally verified, and the multiple hormone- and stress-related motifs only indicate the possibility that *GhSOS3* transcription is influenced by several regulatory inputs.

The predicted PPI network provides a computational framework for the GhSOS3 family, suggesting possible associations with calcium-signaling proteins, particularly CIPK-type kinases (CIPK8, CIPK9-like, CIPK24 and CIPK24-like). These predictions are in line with the CBL–CIPK system described in other plants [26,30], but they do not provide evidence of physical interaction between GhSOS3 proteins and these partners. In particular, the presence of CIPK24-type (SOS2-like) kinases among the predicted partners should not be interpreted as evidence that specific GhSOS3 proteins participate in the canonical SOS pathway, and no SOS1-like protein was among the predicted partners. Several nodes were uncharacterized proteins, and STRING associations integrate indirect evidence such as co-expression, text mining and homology transfer [31,32]. The network is therefore presented only as a hypothesis-generating framework for selecting candidate interactions for experimental testing, for example by yeast two-hybrid, bimolecular fluorescence complementation or co-immunoprecipitation assays. Phosphorylation-site prediction identified several putative sites in the GhSOS3 proteins. In Arabidopsis, phosphorylation of SOS3-like calcium-binding proteins by their interacting SOS2-like kinases is a common regulatory mechanism [33,34]; the predicted sites may therefore represent candidate post-translational regulatory residues. However, sequence-based prediction cannot prove in vivo phosphorylation or identify the responsible kinase [35], and the predicted sites should be considered candidates for experimental verification, for example by phosphoproteomics or site-directed mutagenesis. The structural modeling revealed structural features in the *Gh*SOS3 proteins that are consistent with their sequence relationships. Ramachandran analysis indicated that the predicted models have acceptable stereochemical quality [36,37]; however, this analysis measures the quality of the predicted models [38] and not structural stability under drought, heat, or salinity stress. Similarly, the prediction of EF-hand regions indicates a structural framework that might coordinate calcium but does not prove calcium-binding ability [39]. Thus, experimental assays will be required to test the functionality of the predicted structures. The use of public RNA-seq data along with qRT-PCR showed that *GhSOS3-2* and *GhSOS3-3* were transcriptionally responsive to the abiotic stresses tested. The two genes differed in their levels of expression and timing of transcript accumulation upon stress treatment. However, differences in expression dynamics do not imply different biochemical or biological roles for *GhSOS3-2* and *GhSOS3-3*, because transcript abundance does not necessarily reflect protein abundance or activity [40]. For example, a relatively earlier increase in *GhSOS3-2* cannot be used as an indicator that it is an early stress sensor. Similarly, the *GhSOS3-3* response cannot be taken as an indication of a special function in stress adaptation or recovery. There was genotype-dependent variability in *GhSOS3* transcript levels, with genotype 1 showing higher levels than genotypes 2 and 3 under the stress conditions examined. This observation indicates that *GhSOS3* transcriptional responses can vary with the genetic background of cotton; however, the regulatory mechanisms underlying this variation were not investigated in the present study. Genotype-dependent variability in the expression of stress-responsive genes has been observed previously in cotton [9,41]. The expression patterns of *GhSOS3-2* and *GhSOS3-3* are consistent with, but do not demonstrate, the involvement of these genes in calcium-mediated stress signaling; functional analysis is necessary to determine whether the transcriptional responses are linked to specific signaling or physiological functions [30,42]. Earlier research on various plant species has demonstrated roles of individual CBL proteins in stress-related signaling through interaction with specific CIPK proteins and regulation of ion transport [42,43]. In cotton, VIGS-based silencing of *GhSOS3* and *GhCBL10* increased salt–alkali sensitivity [21]. GhSOS3-5 (Gohir.D05G044500) was annotated as CBL10-like and, like the previously silenced *GhCBL10* (Gh_D05G0440), is located on chromosome D05; cross-mapping of gene identifiers between genome assemblies will be needed to determine whether these correspond to the same locus. Although these results offer insight into *GhSOS3* genes in cotton, they do not assign specific roles to *GhSOS3-2* or *GhSOS3-3*, nor do they confirm functional similarity between GhSOS3 proteins and CBL/SOS3 proteins in other plant systems.

Overall, this research provides a family level genomic perspective on the *GhSOS3* gene family of *G. hirsutum*. The identification of *GhSOS3* genes as candidates for calcium-related stress-signaling studies is based on several lines of evidence: EF-hand-related motifs, evolutionary relationships with known CBL/SOS3 proteins, synteny among *GhSOS3* genes, predicted regulatory elements, predicted protein associations, predicted structural features, and stress-responsive expression profiles. However, only the expression data are experimental; the remaining analyses are computational predictions. Consequently, the current results cannot be interpreted as proof that individual *Gh*SOS3 proteins possess stress-tolerance activity or functional calcium-binding ability, neither of which has yet been experimentally validated in cotton. Future studies should therefore test the predictions generated here using complementary approaches: subcellular localization assays with fluorescent-protein fusions to determine the actual localization of each GhSOS3 protein; yeast two-hybrid, bimolecular fluorescence complementation or co-immunoprecipitation assays to test the predicted interactions; promoter–reporter and transcription factor-binding assays to evaluate the predicted cis-elements; Ca^2+^-binding assays to test the predicted EF-hand function; and gene silencing, knockout or overexpression combined with complementation to determine whether individual GhSOS3 members contribute to drought, salinity and heat tolerance. These experiments would provide the evidence needed to move from the computationally predicted candidate functions described above to an understanding of the mechanisms of *GhSOS3* stress responses in cotton.

In summary, the results indicate that the *GhSOS3* gene family is conserved and transcriptionally stress-responsive and has several features that merit further study. Rather than assigning biological functions to individual members of the *GhSOS3* family, this work reports experimentally measured expression patterns together with computationally predicted features.

## 4. Materials and Methods

### 4.1. Identification of GhSOS3 from the Database

Salt Overly Sensitive 3 (SOS3) query protein sequences were retrieved from the *Arabidopsis thaliana* database TAIR V.3.2.3 (https://www.arabidopsis.org/) [44] accessed on 5 May 2026. A BLASTP (Tbtools-II v2.476) search was performed against the *Gossypium hirsutum* v3.1 proteome [45] retrieved from Phytozome v13 (https://phytozome-next.jgi.doe.gov/) [46], (accessed on 5 May 2026) and hits with an E-value ≤ 1 × 10^−10^ were selected as candidates for further domain validation. The identified protein sequences were subjected to the NCBI CDD batch search for conserved domains using default parameters [47] and verified using the Pfam database [48] and SMART [49] with default settings. All protein sequences lacking the EF-hand domain were discarded, and only non-redundant proteins containing the characteristic EF-hand domain and showing sequence similarity to the SOS3 query proteins were retained as members of the *G. hirsutum* GhSOS3 family.

### 4.2. Gene Structure, Domain, and Motif Analysis

Gene structures of all putative genes were analyzed with the Gene Structure Display Server GSDS v2.0 (https://gsds.gao-lab.org/) [50] (accessed on 15 May 2026) using CDS and genomic sequences as input data. Domains of all proteins were determined using NCBI CDD domain data and visualized with TBtools-II [51]. Conserved motifs were predicted using the MEME suite (https://meme-suite.org/meme/) [52] (accessed on 15 May 2026) with the following parameters: maximum number of motifs = 10, minimum motif width = 6, maximum motif width = 50. Motif distributions were graphically visualized using TBtools-II.

### 4.3. In Silico Physicochemical Properties and Subcellular Localization of GhSOS3

Protein molecular weights and isoelectric points (pI) were computed using the ExPASy ProtParam tool [53], while genomic coordinates and coding sequence (CDS) lengths were retrieved from Phytozome. Protein subcellular localization was predicted using CELLO v2.5 [54] and WoLF PSORT [55], and the prediction values were displayed as a clustered heatmap (Figure 2).

### 4.4. Chromosomal Mapping of GhSOS3

Chromosomal positions of all identified *GhSOS3* genes on both subgenomes (A and D) were obtained from the Phytozome annotation and visualized using MapGene2Chromosome v2 (MG2C; http://mg2c.iask.in/mg2c_v2.0/) [56] (accessed on 20 May 2026) (Figure S2).

### 4.5. Ka/Ks Calculation and Collinearity Analysis

Ka (non-synonymous) and Ks (synonymous) substitution rates were calculated for the *GhSOS3* gene pairs using KaKs_Calculator 2.0 [57] with the default model, based on codon alignments of the coding sequences generated in MEGA12 [58]. Ka/Ks ratios below, equal to and above one were interpreted as indicating purifying, neutral and positive selection, respectively [28]. Collinearity (synteny) analysis was performed with the MCScanX algorithm [59] implemented in the One Step MCScanX module of TBtools-II (version 2.476) [51]. The whole-genome protein sequences and the GFF3 annotation file of *G. hirsutum* v3.1 were used as input. An all-against-all BLASTP search was first performed within TBtools-II (E-value ≤ 1 × 10^−10^; maximum of five hits per query), and collinear blocks were then identified by MCScanX with its default parameters (match score = 50; gap penalty = −1; minimum of five collinear gene pairs per block; maximum of 25 intervening genes between adjacent anchors; block E-value ≤ 1 × 10^−5^). *GhSOS3* gene pairs located within the identified collinear blocks were extracted and are referred to as collinear (syntenic) pairs. The chromosomal positions of the *GhSOS3* genes and the links between collinear pairs were displayed with the Gene Location Visualize and Advanced Circos functions of TBtools-II (Figure 3a,b) [60]. Collinear relationships identified in this way were used as evidence of positional conservation only; no conclusion about the duplication mechanism was drawn from these data alone.

### 4.6. Phylogenetic Inference

The full-length amino acid sequences of the five GhSOS3 proteins and 24 CBL/SOS3-related and other reference sequences from different plant species (29 sequences in total; Figure 4) were aligned with ClustalW [61] implemented in MEGA12 [58] using default parameters. A phylogenetic tree was then inferred in MEGA12 by the Maximum Likelihood method with the Jones–Taylor–Thornton (JTT) amino acid substitution model [62]. Branch support was evaluated by bootstrap analysis with 1000 replicates [63]. The final dataset comprised 780 positions, and the tree with the highest log-likelihood (−13,224.85) is shown in Figure 4.

### 4.7. Cis-Regulatory Elements Analysis

Promoter sequences (2000 bp upstream region) of the *GhSOS3* genes were analyzed with the PlantCARE database (https://plantpan.itps.ncku.edu.tw/plantpan5/promoter_analysis.php (accessed on 20 May 2026)) [64,65] to identify cis-regulatory elements, and the results were graphically visualized using TBtools-II.

### 4.8. Prediction of Protein–Protein Interactions, Phosphorylation Sites and 3D Structures of GhSOS3 Proteins

Protein–protein interactions of GhSOS3 proteins were predicted using STRING (https://string-db.org/) [31] with *G. hirsutum* as the reference organism, and the network nodes were annotated using the STRING and NCBI gene descriptions (Table S3). Phosphorylation sites (serine, threonine and tyrosine) were predicted using NetPhos 3.1 (https://services.healthtech.dtu.dk/services/NetPhos-3.1/ (accessed on 20 May 2026)) [35,66] with the default threshold of 0.5. To predict the tertiary structures, the amino acid sequences of the five GhSOS3 proteins were submitted to AlphaFold 3 [38], and the model with the highest pLDDT score was retained for each protein. For comparison, the five models were superimposed onto the GhSOS3-1 model in PyMOL (version 2.5; Schrödinger, LLC., New York, NY, USA) with the align command so that all models are displayed in the same orientation. EF-hand motifs and calcium-binding loop residues were annotated manually in PyMOL based on conserved domains identified by NCBI-CDD and Pfam; the canonical Ca^2+^-coordinating positions (e.g., Asp and Glu side chains and backbone carbonyl oxygens in the EF-hand loops) were highlighted. Stereochemical quality was assessed by Ramachandran plot analysis of the backbone φ/ψ dihedral angles [67,68], and hydrogen bonding and solvent accessibility were analyzed with VADAR (http://vadar.wishartlab.com/ (accessed on 20 May 2026)) [69].

### 4.9. GhSOS3 Gene Expression Data Retrieval

Publicly available RNA-seq data for all five *GhSOS3* genes were obtained from BioProject PRJNA490626 [70] through the NCBI database [71] for three abiotic conditions, i.e., drought (20% *w*/*v* PEG), salt (250 mM NaCl), and heat (45 °C) stress, together with the control. FPKM values were log2-transformed and displayed as a heatmap (Figure S3).

### 4.10. Plant Material, RNA Extraction and cDNA Synthesis

Three different genotypes of *G. hirsutum* (CIM-608 (G1), MNH-886 (G2), and VH-295 (G3)) were acquired from the cotton germplasm bank. The seeds were sterilized with 70% ethanol for 1 min, followed by 1% sodium hypochlorite for 5 min, and were rinsed three times with sterile distilled water. The seeds were germinated in containers containing a mixture of peat moss and perlite (3:1) under controlled conditions in a growth chamber: 28/22 °C day/night temperature; 16 h photoperiod; 60–65% relative humidity; and light intensity of 400 μmol m^−2^ s^−1^. Three-week-old seedlings were exposed to stress treatments. Total RNA was extracted from 100 mg of fresh leaves of three-week-old uniform seedlings of the three genotypes using the TRIzol reagent method (Invitrogen, Carlsbad, CA, USA) followed by a manual protocol [72]. Three biological replicates were maintained per treatment per time point, with each replicate consisting of pooled leaves from three individual plants. RNA was precipitated with 500 µL isopropanol from the aqueous phase, and the pellet was air-dried and dissolved in RNase-free water. A NanoDrop spectrophotometer (Thermo Fisher Scientific, Wilmington, DE, USA)was used to assess RNA purity and concentration [73], and integrity was confirmed by 1% agarose gel electrophoresis. cDNA was synthesized with the RevertAid First Strand cDNA Synthesis Kit (Thermo Fisher Scientific, Waltham, MA, USA) [74] using a mix of oligo(dT) primers and random hexamers. Diluted cDNA was stored at −80 °C until qPCR analysis.

### 4.11. Gene Expression Analysis (qPCR) of GhSOS3

The gene-specific primers and the internal control gene are given in Table S1. Expression of *GhSOS3* genes was analyzed using the Fast SYBR Green qPCR system with SYBR Green ReadyMix. The amplification protocol comprised denaturation, annealing, and extension steps. Gene expression levels were calculated for each replicate under drought (20% *w*/*v* PEG), salt (250 mM NaCl), and heat (45 °C) stresses. Each biological replicate (*n* = 3) was assessed with three technical replicates, along with a no-template control (NTC) in place of cDNA for each primer pair. Relative expression levels were calculated using the 2^−ΔΔCt^ method [75], with *GhUBQ14* as the reference gene and the 0 h control as the calibrator. The data are shown as mean ± standard error of the mean of the three biological replicates. The significance of the results was determined by three-way ANOVA with genotype, stress treatment, and time as the main factors, including all two-way and three-way interaction effects, and Tukey’s honestly significant difference (HSD) test was used as the post hoc multiple-comparison test at *p* < 0.05. Graphs were plotted using GraphPad Prism software version 9.0 (GraphPad Software Inc., La Jolla, CA, USA), with asterisks used to denote statistically significant differences (* *p* < 0.05, ** *p* < 0.01).

## 5. Conclusions

This study presents a family level in silico genome-wide analysis of the *GhSOS3* gene family in *G. hirsutum*, identifying five genes that encode EF-hand-containing proteins of the SOS3/CBL family. The analysis revealed structural diversity, predicted subcellular localization, predicted stress- and hormone-related cis-regulatory elements, and predicted interaction partners among GhSOS3 proteins. The *GhSOS3* genes showed unequal distribution between the A and D subgenomes, collinear relationships among four of the five genes, and Ka/Ks ratios below one, consistent with purifying selection; however, the mechanism underlying their duplication was not established and requires further synteny-based and phylogenetic investigation. Experimentally, qPCR showed that *GhSOS3-2* and *GhSOS3-3* transcripts accumulated in a stress-, time- and genotype-dependent manner under salt, drought and heat stress. The computational analyses identify candidate structural, regulatory and interaction features that are useful for generating hypotheses about GhSOS3 function, but they do not demonstrate calcium binding, phosphorylation, pathway participation or stress tolerance. These findings provide a genomic and expression-based framework for further functional investigation of the *GhSOS3* gene family in cotton; they should be interpreted cautiously, as the functional roles of the *GhSOS3* genes have not been experimentally validated in the present study.

## Figures and Tables

**Table 1:** In silico physicochemical properties of *Gh*SOS3 proteins. Chr, chromosome; Forward/Reverse, DNA strand; CDS, coding sequence; MW, molecular weight; pI, isoelectric point.

Transcript ID	Gene Name	Chr. NO	Start	End	Strand	CDS (bp)	Protein MW (kDa)	Protein Length (AA)	pI
Gohir.A01G093800.3.p	*GhSOS3-1*	A01	14454840	14457987	Forward	681	25.98	226	4.77
Gohir.A03G003700.1.p	*GhSOS3-2*	A03	972162	975896	Reverse	642	24.33	213	4.74
Gohir.A04G005800.2.p	*GhSOS3-3*	A04	597584	600982	Forward	681	25.95	226	4.96
Gohir.A11G029200.3.p	*GhSOS3-4*	A11	2487308	2490147	Forward	642	24.43	213	4.72
Gohir.D05G044500.1.p	*GhSOS3-5*	D05	3597432	3600278	Reverse	786	30.12	261	4.89

## Data Availability

RNA-seq data for *GhSOS3* FPKM values can be retrieved from the publicly available cotton transcriptome dataset (NCBI BioProject: PRJNA490626) [70]. All other data supporting the findings of this study are included in the article and its Supplementary Materials.

## Supplementary Materials

The following supporting information can be downloaded at https://www.mdpi.com/article/10.3390/ijms27219561/s1.
