# Supplementary material for: Genome-Wide Identification and In Silico Characterization of the Salt Overly Sensitive 3/Calcineurin B-like (GhSOS3) Gene Family in Upland Cotton (Gossypium hirsutum L.) and Expression Profiling of Two Members Under Drought, Salt and Heat Stress
Source: Int J Mol Sci. 2026 Oct 26;27(21):9561. doi: 10.3390/ijms27219561 (PMC13762603; doi:10.3390/ijms27219561)
Supplement: Supplementary file 1 [file ijms-27-09561-s001.zip › ijms-4500617-supplementary.pdf]

Supplementary Figures

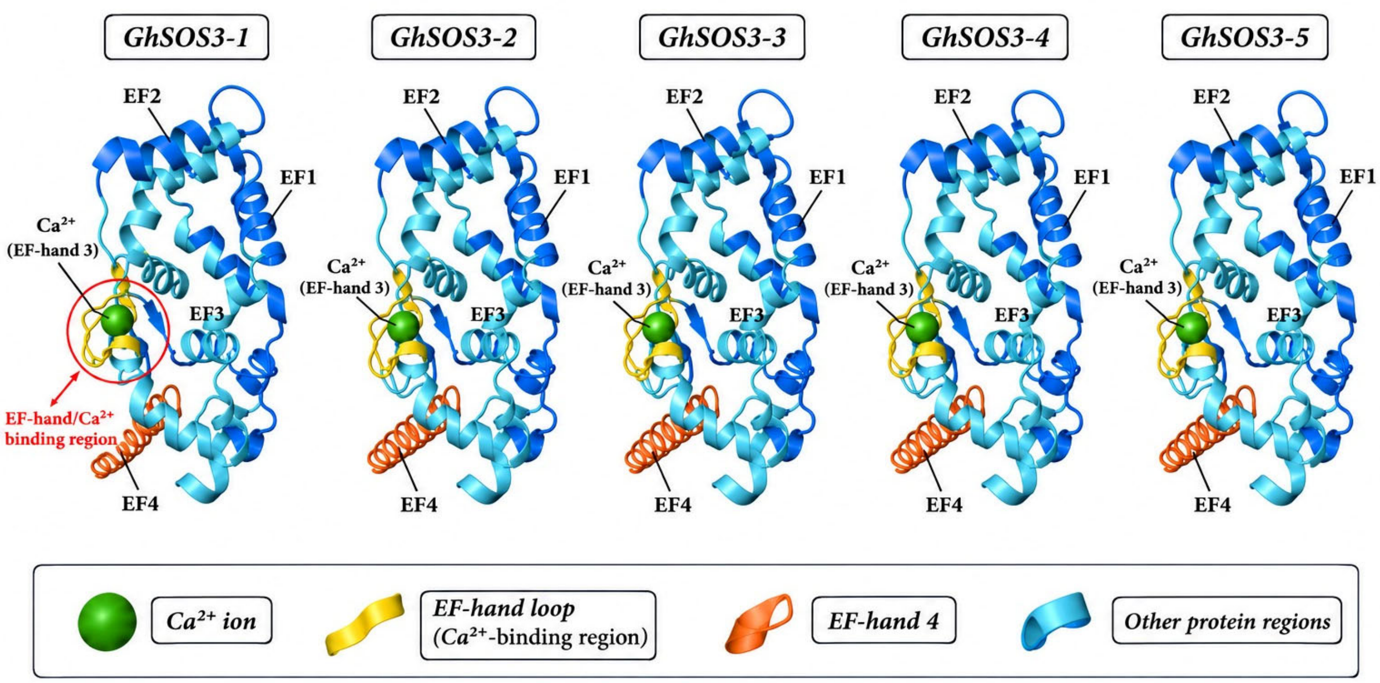

Figure S1. (**A**) Predicted three-dimensional structures of the five GhSOS3 proteins (GhSOS3-1 to GhSOS3-5) generated with AlphaFold 3. All models were superimposed onto GhSOS3-1 in PyMOL and are shown in the same orientation, and each model is labeled. EF-hand motifs EF1–EF4 are shown in different colours (EF1, blue; EF2, green; EF3, orange; EF4, magenta), the 12-residue Ca^{2+}-binding loop of each EF-hand is shown in yellow with the Asp/Glu side chains at the canonical Ca^{2+}-coordinating positions displayed as sticks, and the remainder of each protein is shown in grey. EF-hand positions are based on NCBI-CDD/Pfam annotation; calcium binding was not tested experimentally.

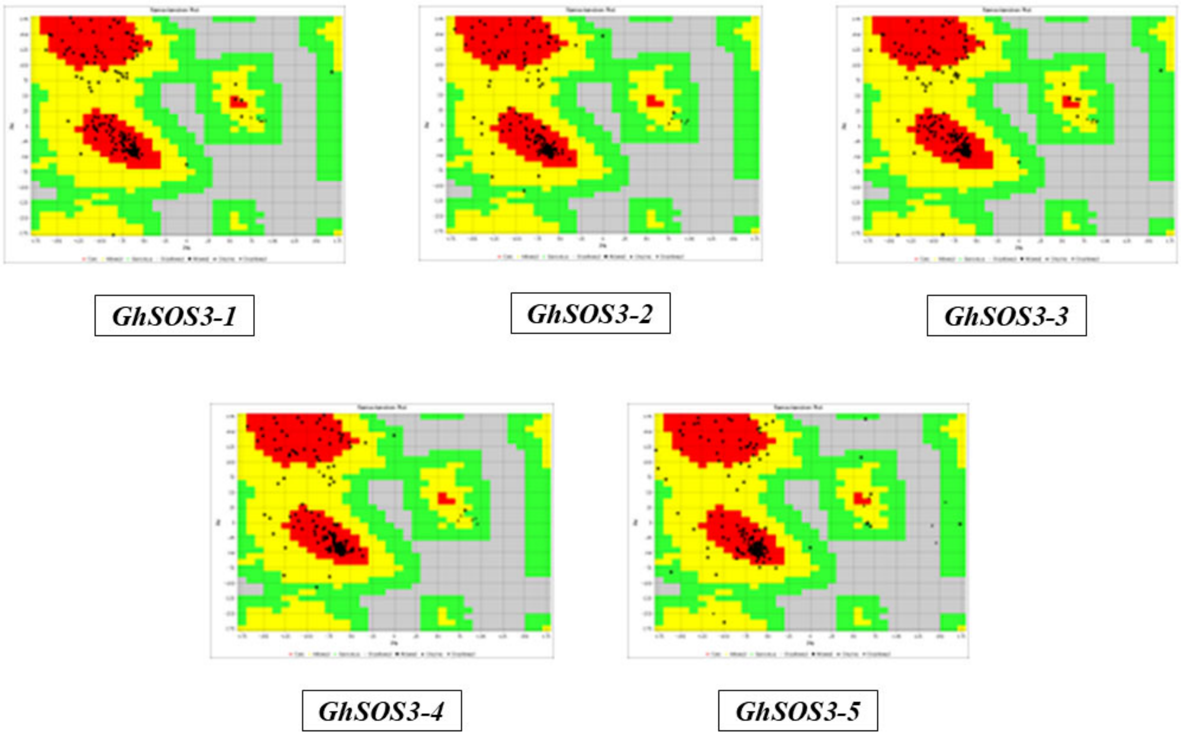

(**B**) Ramachandran plots assessing the stereochemical quality of the predicted GhSOS3 protein models. The majority of residues were located in the most favored and allowed regions, indicating acceptable stereochemical quality of the predicted 3D structures. These results support the use of the structural models for computational analysis, but do not demonstrate functional activity or structural stability under stress conditions.

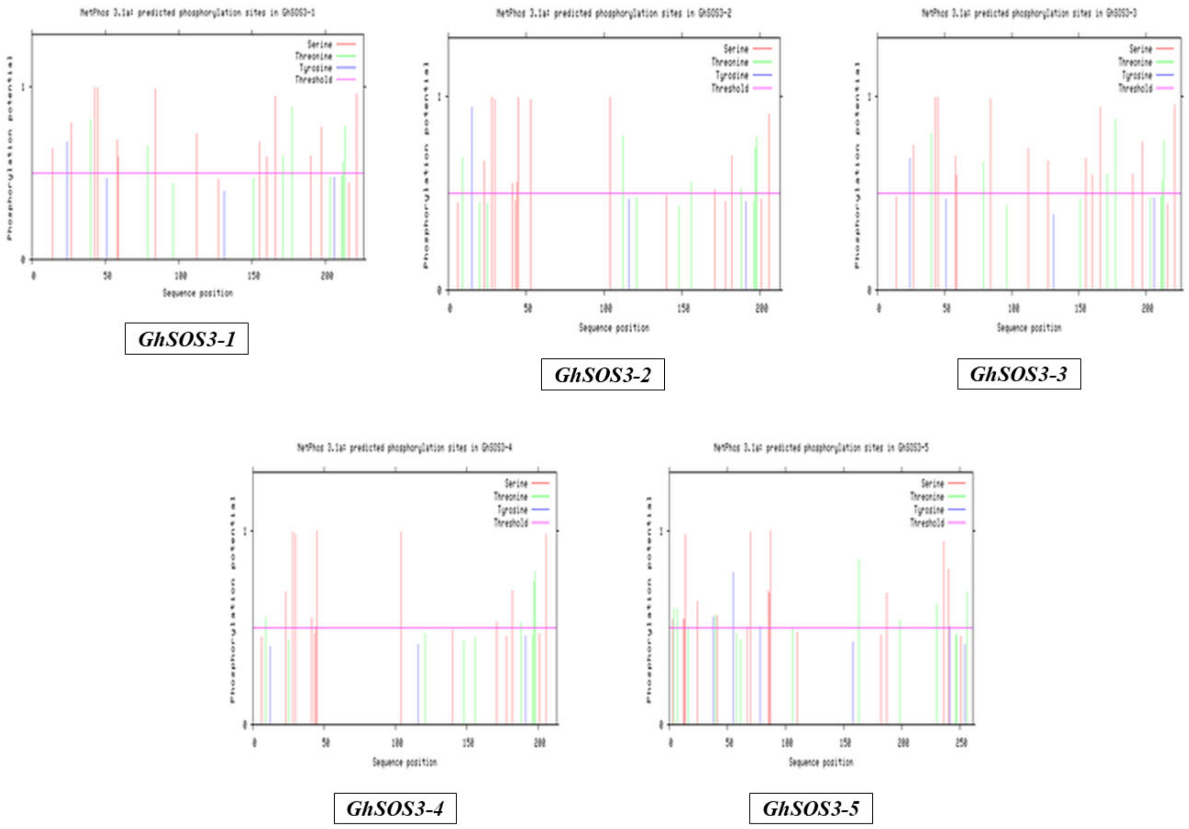

(**C**) Prediction of potential phosphorylation sites in GhSOS3 proteins with NetPhos 3.1. Multiple serine (red), threonine (green), and tyrosine (blue) phosphorylation sites were computationally identified; bars above the threshold line (0.5, pink) indicate predicted sites. These are predicted post-translational modification sites requiring experimental validation.

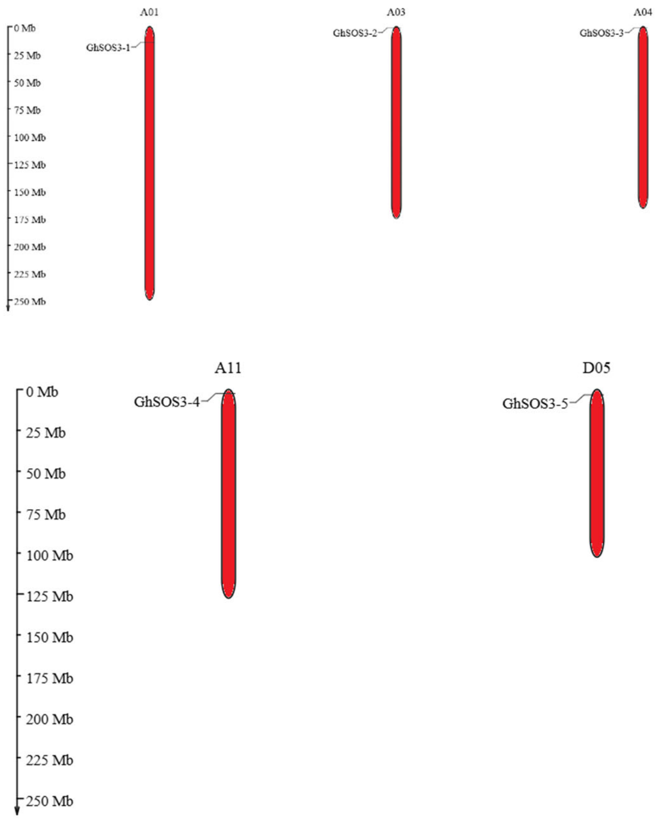

Figure S2. Chromosomal localization of *GhSOS3* genes on chromosomes A01, A03, A04, A11 and D05 of the *G. hirsutum* A and D subgenomes. The genes were unevenly distributed between the two subgenomes. The chromosomal map was created with the online tool MG2C v2.0; the scale bar indicates physical position (Mb).

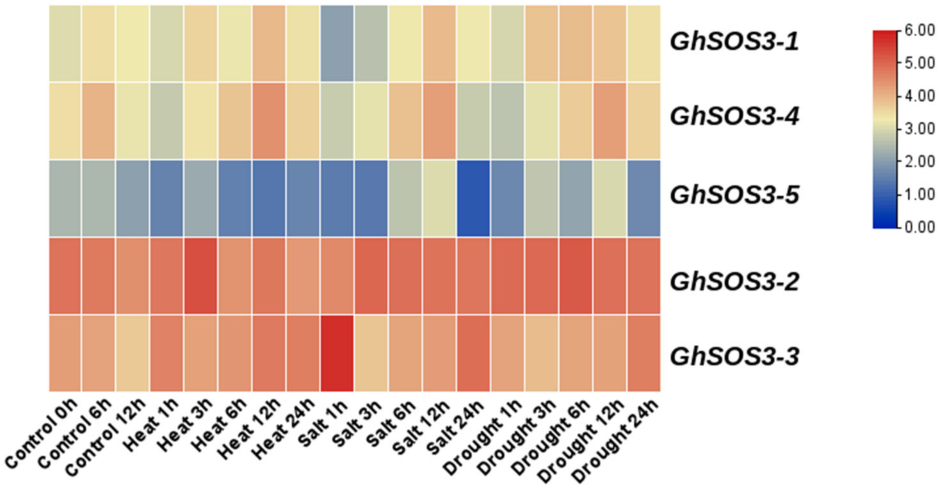

Figure S3. Heatmap of *GhSOS3* genes showing log2-transformed FPKM values from public RNA-seq data (BioProject PRJNA490626) under control, heat, salt, and drought stress conditions (0–24 h). The colour scale represents log2(FPKM) values (0–6). *GhSOS3-2* and *GhSOS3-3*, which showed the highest overall expression across conditions, were selected for qRT-PCR analysis.

Supplementary Tables:

Table S1. Primers used for qPCR analysis.

Primers for expression study			
**SR. No**	**Transcripts/Genes**	**Forward primer (5′ to 3′)**	**Reverse primer (5′ to 3′)**
1	*GhUBQ14* (Housekeeping)	CAACGCTCCATCTTGTCCTT	TGATCGTCTTTCCCGTAAGC
2	*GhSOS3-2*	GCTGATGAAACCATCGAGGC	CCTGAGGTATGGGAGGGTCA
3	*GhSOS3-3*	TGCGGTGATAGATGATGGGC	GGGTGCATTCGGATGGAAGA

Table S2. Ka/Ks values of *Gh*SOS3 gene pairs. Ka, non-synonymous substitution rate; Ks, synonymous substitution rate. Ks values > 2.0 may indicate saturation of synonymous sites, and the corresponding Ka/Ks ratios should be interpreted with caution. Collinear pairs were identified with MCScanX (TBtools-II); collinearity indicates a syntenic relationship and does not by itself establish the duplication mechanism.

Paralog 1	Paralog 2	Ks	Ka	Ka/Ks	Collinearity (MCScanX)	Selection (Ka/Ks < 1)
Gohir.A01G093800.3 (*GhSOS3-1*)	Gohir.A03G003700.1 (*GhSOS3-2*)	2.028	0.272	0.134295	Collinear pair	Purifying
Gohir.A01G093800.3 (*GhSOS3-1*)	Gohir.A04G005800.2 (*GhSOS3-3*)	0.446	0.008	0.01806	Collinear pair	Purifying
Gohir.A01G093800.3 (*GhSOS3-1*)	Gohir.A11G029200.3 (*GhSOS3-4*)	2.215	0.277	0.124865	Collinear pair	Purifying
Gohir.A03G003700.1 (*GhSOS3-2*)	Gohir.A04G005800.2 (*GhSOS3-3*)	2.330	0.284	0.121707	Collinear pair	Purifying
Gohir.A03G003700.1 (*GhSOS3-2*)	Gohir.A11G029200.3 (*GhSOS3-4*)	0.533	0.041	0.076758	Collinear pair	Purifying
Gohir.A04G005800.2 (*GhSOS3-3*)	Gohir.A11G029200.3 (*GhSOS3-4*)	2.338	0.280	0.119887	Collinear pair	Purifying

Table S3. Annotation of nodes in the *Gh*SOS3 protein–protein interaction network. Node labels correspond to those in Figure 6 of the main text. Annotations are taken from STRING/NCBI descriptions and are predicted, not experimentally verified.

Node	Gene name	Functional annotation
GhSOS3-1	GhSOS3-1 (CBL3)	STRING annotation: calcineurin B-like protein 3-like; predicted (putative) calcium sensor
GhSOS3-2	GhSOS3-2 (CBL1)	STRING annotation: calcineurin B-like protein 1-like; predicted (putative) calcium sensor
GhSOS3-3	GhSOS3-3 (CBL2)	STRING annotation: calcineurin B-like protein 2-like; predicted (putative) calcium sensor
GhSOS3-4	GhSOS3-4 (CBL1)	STRING annotation: calcineurin B-like protein 1-like; predicted (putative) calcium sensor
GhSOS3-5	GhSOS3-5 (CBL10)	STRING annotation: calcineurin B-like protein 10-like; predicted (putative) calcium sensor
LOC107901302	CIPK24: CBL-interacting serine/threonine-protein kinase 24	CBL-interacting serine/threonine-protein kinase 24; predicted role in calcium-mediated signal transduction
LOC107908757	UP1: Uncharacterized protein	Uncharacterized protein LOC107908757
LOC107927946	UP2: Uncharacterized protein	Uncharacterized protein LOC107927946

LOC107933416	UP3: Uncharacterized protein	Uncharacterized protein LOC107933416
LOC107936805	RAD4	DNA repair protein RAD4; annotated role in DNA damage recognition and repair
LOC107939873	UP4: Uncharacterized protein	Uncharacterized protein LOC107939873
LOC107941797	PK: Protein kinase	Non-specific serine/threonine-protein kinase belonging to the protein kinase superfamily
LOC107942684	CIPK9-like	CBL-interacting serine/threonine-protein kinase 9-like; predicted role in CBL-mediated calcium signaling
LOC107957846	CIPK8: CBL-interacting serine/threonine-protein kinase 8	CBL-interacting serine/threonine-protein kinase 8; predicted role in CBL-mediated calcium signaling
LOC107962610	CIPK24-like: CBL-interacting serine/threonine-protein kinase 24-like	CBL-interacting serine/threonine-protein kinase 24-like; predicted role in CBL-mediated calcium signaling
